# Prevalence, age of decision, and interpersonal warmth judgements of childfree adults

**DOI:** 10.1038/s41598-022-15728-z

**Published:** 2022-07-25

**Authors:** Zachary P. Neal, Jennifer Watling Neal

**Affiliations:** Psychology Department, Michigan State University, East Lansing, USA

**Keywords:** Human behaviour, Epidemiology

## Abstract

Childfree adults do not want to have children, making them distinct from parents and other adults without children. However, they are difficult to study because they cannot be identified using conventional data on fertility. We use data from a representative sample in the United States to examine the prevalence, age of decision, and interpersonal warmth judgments by and about childfree adults. Our prevalence estimates suggest that childfree adults are quite common, comprising over one-fifth (21.64%) of the population. Our analysis of age-to-decision suggests that most childfree adults reported that they decided they did not want children early in life. Finally, our analysis of interpersonal warmth suggests asymmetric affective polarization among parents and childfree adults driven primarily by parent’s ingroup favoritism. We discuss the implications of these findings for our understanding of childfree adults and for future research on this historically overlooked segment of the population.

## Introduction

In the United States and other Western industrialized countries, the majority of adults eventually become *parents*. However, fertility rates have declined^1–4^ and fewer adults expect to have children during their lifetime than in the past^5,6^. Many of these adults simply do not want children^6^ and are characterized as *childfree* in the popular press^7–9^ and academic literature^10–12^. Childfree adults are distinct from *childless* adults who wanted children but were unable to have them, and from *not-yet-parents* who are planning to have children in the future^13^. Because childfree adults explicitly do not want children, they are also different from adults who are *undecided* about whether they plan to have children, and from adults who do not plan to have children but are *ambivalent* or indifferent about whether they wanted children.

Understanding childfree adults is important because they may make up a sizeable portion of the population^13^, and because declining fertility rates suggest that the number of childfree adults may be growing^1–6^. Members of this large population experience unique barriers in the workplace and healthcare. For example, childfree adults are often neglected in discussions of work-life balance^14,15^ and are commonly denied access to voluntary sterilization by their physicians^16^. Childfree adults also experience stigmatization and are the recipients of negative stereotypes from parents and other adults without children^17–27^.

Qualitative studies have yielded insight into the experiences of childfree adults^14,15,28–32^, while quantitative studies have attempted to estimate the prevalence of childfree adults^13,33–35^ and measure attitudes toward them^13,17–19,21,23–25^. However, many of these past studies are limited in their generalizability because they focus exclusively on women^33,34^ or rely on non-representative samples^14,15,17,21,24,28–31^. In this work, to be inclusive we examine the entire population, including both men and women, but report results separately for men and women in the *Supplementary Information*. Research on childfree adults that involves representative samples is challenging because few large-scale surveys directly measure whether respondents want to have children. Without such a direct measure, studies risk conflating childfree adults with other adults that do not have children, or inappropriately rely on biological indicators (e.g., fertility, or whether one is *able* to have children) to measure a non-biological status (i.e., whether one *wants* to have children)^13,33^. Indeed, infertile individuals may still identify as childfree if they explicitly do not want children. Additionally, infertile individuals who want children may still become parents through adoption or step-parenting.

These challenges have made it difficult to determine how many adults are childfree. Past estimates of the prevalence of childfree adults are typically low, ranging from 2.2% to 9%^33,35,36^. However, these estimates focused exclusively on women and used fertility-based measures, which may yield underestimates. More recent work that included men and directly measured of respondents’ desire to have children estimated a substantially higher prevalence of 27%^13^. In this paper, we use data from a large ($N = 1500$) representative sample of adults in Michigan to estimate the prevalence of childfree adults.

A life-course perspective implies that individuals may change from planning to have children or being ambivalent about having children to childfree over time^37^. Recognizing that many individuals decide not to have children, some research has sought to understand when they reach this decision. The existing literature suggests that people fall into two camps with respect to when they decide to become childfree^11,38–40^. *Early articulators* decide to become childfree when they are quite young, often before marriage or partnership, and were estimated to account for only one-third of childfree adults in the 1970s^39^. In contrast, most childfree adults were estimated to be *postponers*^39^, who come to the decision to be childfree later in life. These individuals may have initially planned to have children or may have been undecided or ambivalent about having children. Postponers often decided to be childfree after delaying parenthood to meet other objectives, and may reach this decision individually or as part of a marriage or partnership. Consistent with this idea, longitudinal work has suggested that some adults switched from wanting to not wanting a child after delaying parenthood^41^. In this paper, to understand whether childfree adults tend to be early articulators or postponers, we use retrospective methods to examine the age at which childfree adults decided they did not want children.

Although some research on childfree adults has focused on issues of prevalence and age, more research has examined perceptions of and feelings toward childfree adults.^17–27^. People perceive childfree adults as having more negative traits and less psychological fulfillment than parents^17,19–24,26,27^. People also express more negative emotions such as moral outrage, pity, or disgust toward childfree adults than parents^17,19^, and parents feel less warm toward childfree adults than childfree adults feel toward each other^13^. Although this work suggests childfree adults occupy a marginalized status relative to parents, it has relied on non-representative college or convenience samples. In this paper, we use a representative sample to examine within- and between-group judgements of interpersonal warmth among parents and childfree adults.

## Results

### Prevalence

**Figure 1:**
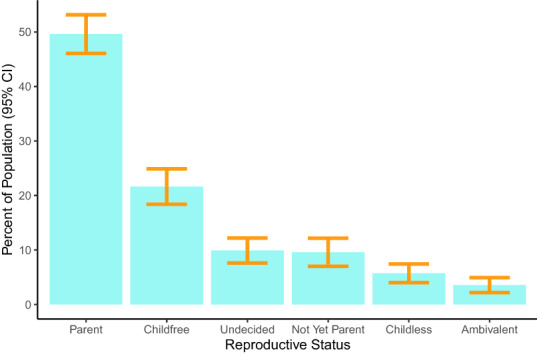
Prevalence of reproductive statuses.

To determine the prevalence of childfree adults, we classified survey respondents into six mutually-exclusive reproductive statuses: parent, childfree, undecided, not-yet-parent, childless, and ambivalent. Figure 1 shows the estimated prevalence of each reproductive status as a percent of the total adult population, with the associated 95% confidence intervals. We find that childfree adults comprise 21.64% (SE = 1.65, 95% CI 18.39–24.88) of the adult population in Michigan. The prevalence of childfree adults is second only to parents who comprise 49.62% (SE = 1.81, 95% CI 46.08–53.17) of the population. The other reproductive statuses are substantially less prevalent: Undecided (9.9%), Not-yet-parents (9.58%), Childless (5.72%), Ambivalent (3.55%). Supplementary Information S1 reports prevalence by gender subgroups, which revealed few differences.

These prevalence estimates describe the entire adult population of Michigan. However, decisions about whether or not to have children are particularly pressing and salient for women without children under age 40. In this critical subpopulation, most (36.71%, SE = 4.96, 95% CI 26.99–46.43) report planning to have children (i.e., are not-yet-parents). However, we find that more report not wanting to have children (30%, SE = 4.46, 95% CI 21.25–38.75) than being undecided (26.8%, SE = 4.29, 95% CI 18.39–35.22).

### Age of decision

**Figure 2:**
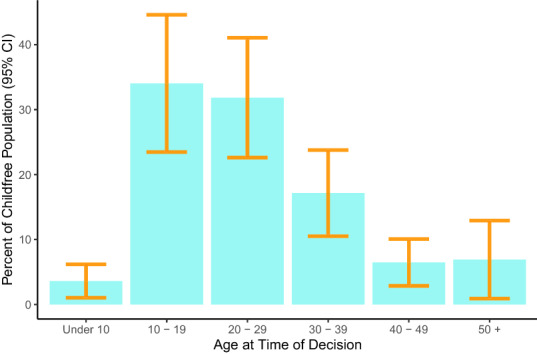
Age when childfree adults report that they decided to be childfree.

To determine when people decided to be childfree, we asked each survey respondent classified as childfree in what decade of life they reached this decision. Figure 2 shows the percent of the childfree population reporting that they decided they did not want children in each decade of life, with the associated 95% confidence intervals. We find that most childfree adults report that they decided they did not want children during prime childbearing years, in their teens (34.04%, SE = 5.39, 95% CI 23.47–44.61) or twenties (31.84%, SE = 4.71, 95% CI 22.61–41.07). Fewer childfree adults report that they arrived at this decision later in life, in their thirties (17.14%), forties (6.46%), or later (6.91%), while a small percentage of childfree adults report that they knew before age 10 that they did not want children (3.6%). Supplementary Information S2 reports age of decision by gender subgroups, which revealed no differences.

One common response to a woman’s decision not to have children is that she will ‘change her mind’^16,32^. If childfree women did often change their mind and later become parents, we would expect that currently childfree women who decided early not to have children (i.e., early articulators) would be relatively young. However, we find that the average age of women who reported deciding to be childfree before age 20 is 38.58 (SE = 3.57). That is, on average, we observe that early articulator women are older and report that they made the decision to be childfree at least 18 years ago.

### Interpersonal warmth judgements

**Figure 3:**
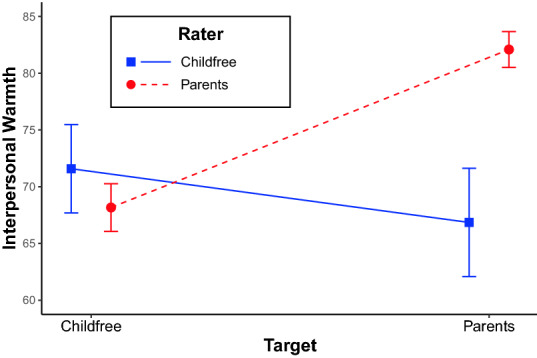
Interpersonal warmth felt by childfree adults and parents toward each other.

To examine within- and between-group judgements of interpersonal warmth among parents and childfree adults, we asked both parent and childfree respondents how warmly they felt toward each other. Figure 3 summarizes the mean interpersonal warmth judgements of parents (dashed red line) and childfree adults (solid blue line), with the associated 95% confidence intervals. Supplementary Information S3 reports interpersonal warmth judgements by gender subgroups, which revealed few differences.

First, we find that parents feel significantly warmer toward parents ($\hbox {M} = 82.09$, $\hbox {SE} = 0.81$) than toward childfree adults ($\hbox {M}=68.17$, $\hbox {SE}=1.07$; $\hbox {t}(896)=-12.63$, $\hbox {p} <0.001$) In contrast, childfree adults exhibit no significant difference in warmth felt toward parents ($\hbox {M}=66.85$, $\hbox {SE}=2.44$) and childfree adults ($\hbox {M}=71.58$, $\hbox {SE}=1.98$; $\hbox {t}(231)=1.9$, $\hbox {p} = 0.058$). That is, we observe ingroup favoritism among parents, but not among childfree adults.

Second, we find that parents feel significantly warmer toward parents than do childfree adults ($\hbox {t}(1129) = -5.94$, $\hbox {p} <0.001$). However, parents and childfree adults feel similar levels of warmth toward childfree adults ($\hbox {t}(1129) =1.52$, $\hbox {p} = 0.13$). That is, we observe that people are polarized in their feelings toward parents, but not in their feelings toward childfree adults.

Finally, we find that parents feel more ingroup warmth than childfree adults ($\hbox {t}(1129)=-\,4.91$, $\hbox {p}<0.001$). In contrast, parents and childfree adults feel similar levels of outgroup warmth ($\hbox {t}(1129)=-\,0.49$, $\hbox {p}=0.621$). That is, we observe that group differences in interpersonal warmth are driven by parents’ ingroup favoritism.

## Discussion

Our analysis of prevalence suggests that childfree adults are or have become more common in the US than previously thought. Childfree adults comprised over one-fifth (21.64%) of the adult population in Michigan and were second in number only to parents. This means that childfree adults were the single largest group of adults without children, exceeding not-yet-parents as well as childless, undecided, and ambivalent adults. Moreover, we find that the prevalence is even higher (30%) among women of childbearing age who do not have children. These prevalence estimates are consistent with one recent estimate (27%) obtained using desire-based measures^13^, but are substantially higher than past estimates (2.2–9%) obtained using fertility-based measures^33,35,36^. There are multiple possible explanations for this discrepancy, including that past estimates systematically undercounted childfree adults by excluding infertile adults who did not want children, and that the prevalence of childfree adults has grown over time. In either case, our findings suggest that a large proportion of the adult population do not have children and do not want them. This is important because it suggests the size of this population has been underestimated, which may have implications for demographic and political trends. For example, although US politicians often develop policies designed to support parents and children^42^, they rarely focus on the needs of the large and potentially growing population of childfree adults.

Our analysis of age-to-decision suggests that the majority of childfree adults report that they are early articulators who decided in their teens or twenties that they did not want children, rather than postponers who decided that they did not want children much later in life. This represents a shift in decision-making from the 1970s when only one-third of childfree adults were estimated to be early articulators^39^. We also observe that the women who reported deciding in their teens and twenties that they did not want children are now in their late-thirties on average. This means that many of these women recall having made the decision to be childfree nearly two decades ago. The large number of early articulators, together with women’s reported persistence in their decision not to have children, could point to changing norms toward parenthood and increasing recognition of the childfree choice as a viable alternative^7–9,12^. This is important because it suggests that when doctors greet “childfree women’s requests for sterilizations with hesitation [due to] fear that a woman will change her mind later”^16^, it is misinformed and paternalistic.

Our analysis of interpersonal warmth suggests affective polarization among parents and childfree adults, such that these groups exhibit significant differences in their feelings of warmth toward each other. However, these differences are not equal. We observe that the warmth parents feel toward other parents is particularly strong, which highlights that this polarization is asymmetric. Recent research on asymmetric affective polarization in US politics, which traces its “origins to the power of partisanship as a social identity”^43^, may offer some insight. The warmth differences we observe may be driven by parenthood as a social identity that is highly visible. The asymmetry in warmth differences may stem from the fact that choosing not to have children has not been a salient or publicly-disclosed social identity. However, the recent growth of books^7^, films^8^, and online communities^32^ focused on childfree adults may point to childfree-ness emerging as a distinctive social identity, which could serve to widen these differences in the future.

This research has several strengths that make important contributions to our understanding of US adults’ reproductive decisions. First, it uses a more nuanced classification of reproductive status than prior studies that shifts the focus from biology (e.g., ability to have children) to behavior (e.g., desire to have children). Second, it is among the largest representative-sample studies of childfree adults, and therefore offers greater generalizability than prior studies relying on convenience and non-representative samples. The findings also have important implications for US demography and reproductive freedom. First, they highlight the potential for continuing and accelerating declines in fertility rates, which may be accompanied by new household structures and new ways of ‘starting a family’ that do not involve having children. Second, by revealing the large number of adults who have decided not to have children, and their long-term persistence in this decision, it highlights for undecided and ambivalent adults that the childfree choice is a common alternative to parenthood.

These findings are subject to some limitations, which also highlight directions for future research. First, these data come from one US state (Michigan), albeit one that is both politically and demographically similar to the US as a whole. Thus, the findings require replication in other states and world regions. Second, these data were collected in Fall 2021, during the COVID-19 pandemic, which may have impacted respondents’ views about whether they want to have children. However, it seems likely that COVID-19 and other forms of uncertainty (e.g., climate change^44,45^) will continue to have similar effects in the future. Third, these data are cross-sectional, which limits our ability to examine reproductive decision-making across the life course. In particular, there is the risk of survival bias in identifying childfree adults because we only observe adults who were childfree at the time of the survey, but not those who formerly were childfree but later became parents. To overcome this limitation and better understand reproductive decision-making, future waves of national panel surveys such as the Panel Study of Income Dynamics may consider supplementing measurements of fertility with measurements of desire for having children.

This study offers critical insights on childfree adults, who are a demographically significant segment of the population, but whose numbers have been substantially underestimated in the past. We find that one-fifth of the adult population is childfree, and the majority of childfree adults report making the decision not to have children early in life. This means that many people may be at risk of the previously documented negative outcomes experienced by childfree adults, including exclusion from work-life balance considerations, denial of medical care, and attribution of negative stereotypes. Clarifying the size of this population can not only direct attention toward reducing these negative outcomes, but also highlight for young adults that the decision not to have children is quite common.

## Methods

### Sample

We use data from the Michigan State of the State survey, conducted by the Institute for Public Policy and Social Research at Michigan State University in September 2021. The survey included a sample of 1000 adults and an additional 500 parent oversample from Michigan who were matched on gender, age, race, and education to the 2018 American Community Survey. Initial survey weights were computed using propensity scores based on 2020 Presidential vote, age, gender, race/ethnicity, and years of education. These initial weights were then post-stratified on 2016 Presidential vote choice, interest in news, political ideology, born again Christian identification, gender, age, race, and education to obtain the final weights. To ensure the sample is representative of the population, and to reduce sampling error, the results presented in the paper are obtained from the complete weighted $N = 1500$ sample. However, we obtain similar results when using the weighted $N = 1000$ without the parent oversample, and when using the unweighted $N = 1000$ sample. These data are available at http://ippsr.msu.edu/survey-research/state-state-survey-soss/soss-data.

In 2021, Michigan (MI) had an estimated population of 10,050,811. Based on 2021 US Census American Community Survey, its population was demographically similar to the United States as a whole in terms of child population (percent under 18: 21.50% MI, 22.30% US), senior population (percent over 64: 17.70% MI, 16.50% US), race (percent white alone: 79.20% MI, 76.30% US), education (percent with BA: 29.10% MI, 32.10% US), and income (median household: $57,144 MI, $62,843 US). Michigan is also politically similar to the United States (percent voting for Biden in 2020: 50.62% MI; 51.3% US).

### Classification of reproductive status

**Figure 4:**
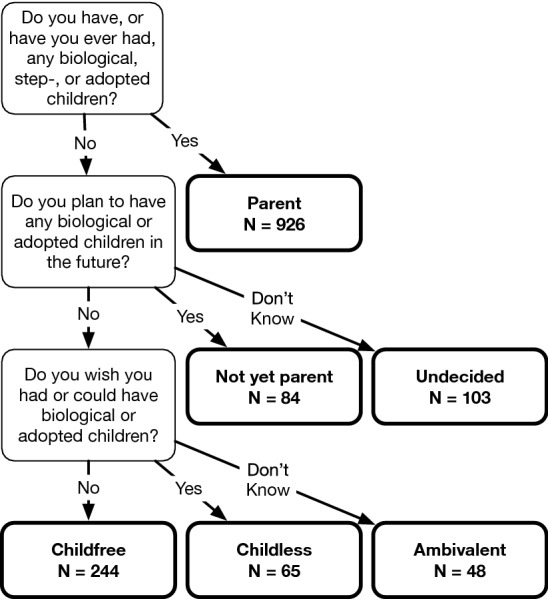
Classification of respondents’ reproductive status.

To classify survey respondents into mutually-exclusive reproductive statuses, we asked up to three binary questions as illustrated in Fig. 4. First, respondents were asked “Do you have, or have you ever had, any biological, step-, or adopted children?” Respondents who answered ‘yes’ were classified as parents ($N = 926$). Respondents who answered ‘no’ were asked “Do you plan to have any biological or adopted children in the future?” Respondents who answered ‘yes’ were classified as not-yet-parents ($N = 84$) and respondents who answered ‘I don’t know’ were classified as undecided ($N = 103$). Respondents who answered ‘no’ were asked “Do you wish you had or could have biological or adopted children?” Respondents who answered ‘yes’ were classified as childless ($N = 65$), respondents who answered ‘I don’t know’ were classified as ambivalent ($N = 48$), and respondents who answered ‘no’ were classified as childfree ($N = 244$). This approach to measuring reproductive status mirrors earlier studies^13^, but allows ‘I don’t know’ response options to the second and third questions, thereby ensuring that undecided and ambivalent adults are not misclassified, which could lead to overestimating the number of childfree adults. Due to missing data, we were unable to classify 30 (2%) respondents, who were dropped listwise from the analysis of prevalance.

### Measurement of age of decision

To understand when survey respondents decided to be childfree, we asked all respondents classified as ‘childfree’: “How old were you when you decided you did not want to have children?” Because we anticipated that most respondents would have difficulty accurately recalling the exact age when they reached this decision, the response options included whole decades: ‘under 10’, ‘10-19 years old’, ‘20-29 years old’, ‘30-39 years old’, ‘40-49 years old’, ‘50 or older’, and ‘I don’t know’. We verified that no respondent provided a response to this question that was inconsistent with their current age. Due to missing data, 60 (24.6%) of childfree respondents were dropped listwise from the analysis of age to decision.

### Measurement of interpersonal warmth

Interpersonal warmth was measured using two feelings thermometer questions: “*On a 0 to 100 scale, where 0 means very cold or unfavorable, and 100 means very warm or favorable, how do you feel toward people who never want to have or adopt children?*” and “*On a 0 to 100 scale, where 0 means very cold or unfavorable, and 100 means very warm or favorable, how do you feel toward people who have children?*” To avoid order effects, these two questions were presented in random order. Due to missing data, 39 (3.3%) of childfree and parent respondents were dropped listwise from the analyiss of interpersonal warmth.

### Analysis

Analyses were conducted in R^46^ version 4.1.2. using the survey package^47^. Because there are six possible within-group and between-group differences in interpersonal warmth that could be tested, we use a Holm-Bonferroni correction to ensure a familywise error rate of $\alpha = 0.05$ across six tests.

### Ethical approval

This study relies on data collected by the Institute for Public Policy and Social Research at Michigan State University, which was approved by the Michigan State University Institutional Review Board. The analyses reported here were performed in accordance with relevant guidelines and regulations.

## Supplementary Information

Supplementary Information.

## Data Availability

The data and code necessary to replicate all results is available at https://osf.io/8avrd/.
