# Supplementary material for: Prevalence, age of decision, and interpersonal warmth judgements of childfree adults
Source: Sci Rep. 2022 Jul 25;12:11907. doi: 10.1038/s41598-022-15728-z (PMC9314368; doi:10.1038/s41598-022-15728-z)
Supplement: Supplementary file 1 — Supplementary Information. [file 41598_2022_15728_MOESM1_ESM.pdf]

Supplementary Information for: "Prevalence, Age of Decision, and Interpersonal Warmth Judgements of Childfree Adults"

Zachary P. Neal and Jennifer Watling Neal

The primary text reports estimates of the prevalence, age of decision, and interpersonal warmth judgements of all childfree adults in Michigan. Here, we repeat each of these analyses separately for respondents who identify as men and as women to consider possible gender differences. These analyses are exploratory and risk being statistically underpowered due to the small sample sizes, and therefore should be interpreted with caution.

S1 Prevalence

Figure S1 shows the prevalence of reproductive statuses in the population of men, while Figure S2 shows their prevalence in the population of women. These figures highlight some potentially notable gender differences. First, more women (54.86% SE = 2.24, 95% CI: 50.47 – 59.25) report being parents than men (44.4% SE = 2.82, 95% CI: 38.87 – 49.93). However, this difference is difficult to interpret because some men may not know whether they are parents. Second, more men (24.86% SE = 2.86, 95% CI: 19.26 – 30.47) report being childfree than women (17.99% SE = 1.69, 95% CI: 14.67 – 21.31). This difference could suggest that men are more likely to be childfree, but could also reflect historically expectations of parenthood have been stronger for women. The prevalence of other reproductive statuses do not differ significantly by gender.

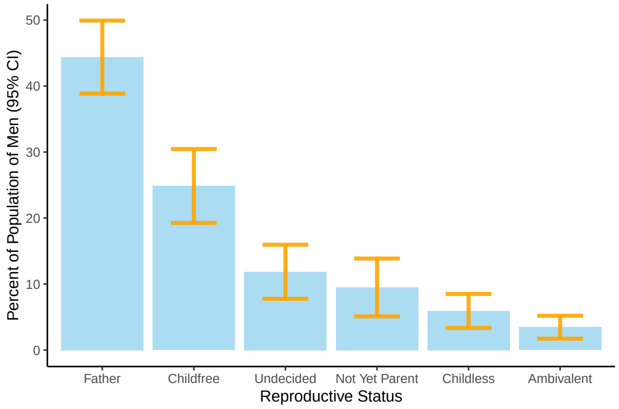

Figure S1: Prevalence of reproductive statuses among men

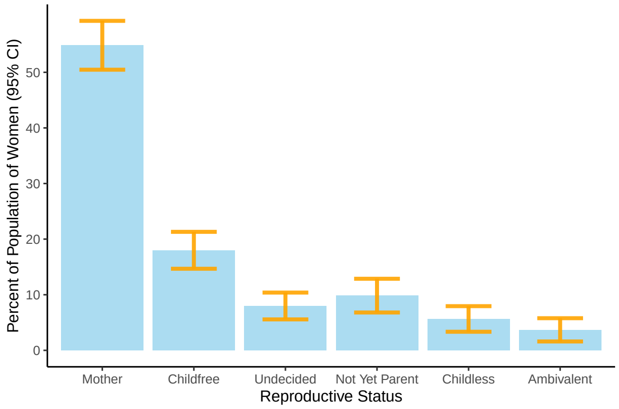

Figure S2: Prevalence of reproductive statuses among women

S2 Age to decision

Figure S3 shows the age at which childfree men decided to be childfree, while Figure S4 shows the age at which childfree women decided to be childfree. We do not observe any significant gender differences in the age at which childfree individuals decided to be childfree.

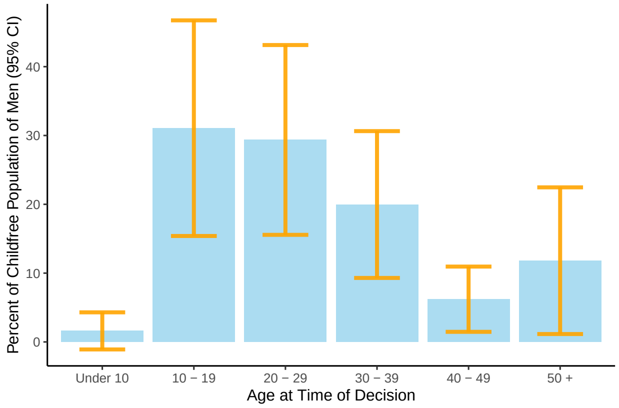

Figure S3: Age when childfree adult men report that they decided to be childfree

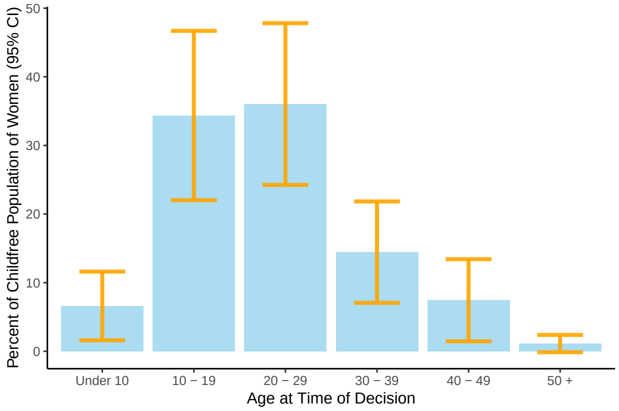

Figure S4: Age when childfree adult women report that they decided to be childfree

S3 Interpersonal warmth

Figure S5 summarizes the mean interpersonal warmth judgements of fathers (dashed red line) and childfree men (solid blue line), with the associated 95% confidence intervals, while Figure S6 summarizes the mean interpersonal warmth judgements of mothers (dashed red line) and childfree women (solid blue line). The pattern observed in the overall population is evident in both gender subgroups: group differences in interpersonal warmth are driven by parents' ingroup favoritism. However, parental ingroup favoritism is somewhat stronger for fathers (warmth toward childfree = 63.91 (1.82), warmth toward parents = 81.68 (1.36), difference = 17.77) than for mothers (warmth toward childfree = 71.16 (1.27), warmth toward parents = 82.36 (0.99), difference = 11.2).

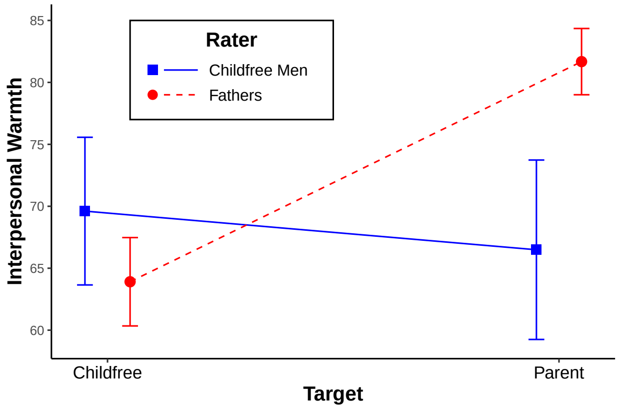

Figure S5: Interpersonal warmth felt by childfree men and fathers toward each other.

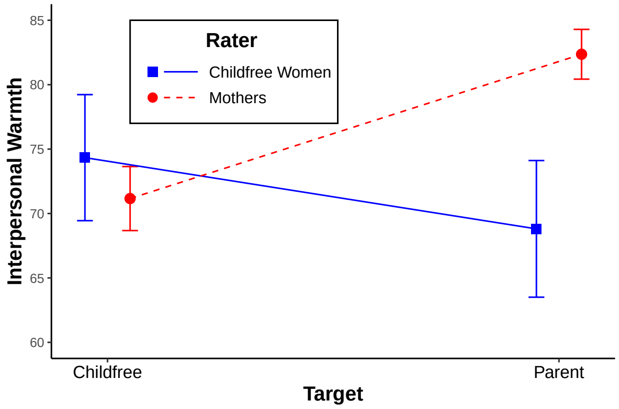

Figure S6: Interpersonal warmth felt by childfree women and mothers toward each other.
